# Musical pitch classes have rainbow hues in pitch class-color synesthesia

**DOI:** 10.1038/s41598-017-18150-y

**Published:** 2017-12-19

**Authors:** Kosuke Itoh, Honami Sakata, Ingrid L. Kwee, Tsutomu Nakada

**Affiliations:** 1 Center for Integrated Human Brain Science, Brain Research Institute, University of Niigata, Niigata, 951-8585 Japan; 2 Department of Neurology, University of California, Davis, USA

## Abstract

Synesthesia, an anomalous blending of senses in which stimulation of one sensory modality produces sensation in a different modality, provides a unique opportunity to study how multimodal information is represented in the human brain. We investigated how pitch classes (*do*, *re*, *mi*, etc.) are associated with the three dimensions of color (hue, saturation, and value/brightness) in 15 subjects who possessed “pitch class-color synesthesia”. Across-subject averaging of reported colors revealed that pitch classes have rainbow hues, beginning with *do*-red, *re*-yellow, and so forth, ending with *si*-violet, accompanied by a decrease in saturation. Enharmonic pitch classes that referred to the same pitch class with a different name produced color sensations according to the name of the base pitch class, e.g., a reddish color for *do-sharp* and a yellowish color for *re-flat*. Thus the main factor producing color sensations was the name, not the sound, of the note; behavioral experiments corroborated this interpretation. Pitch class-color synesthesia represents a newly described type of synesthesia that is distinct from the well-known crossmodal association between pitch height and value/brightness. Findings suggest that the two dimensions of musical pitch, pitch class and pitch height, are mapped to the hue-saturation plane and the value/brightness dimension of color, respectively.

## Introduction

**Figure 1:**
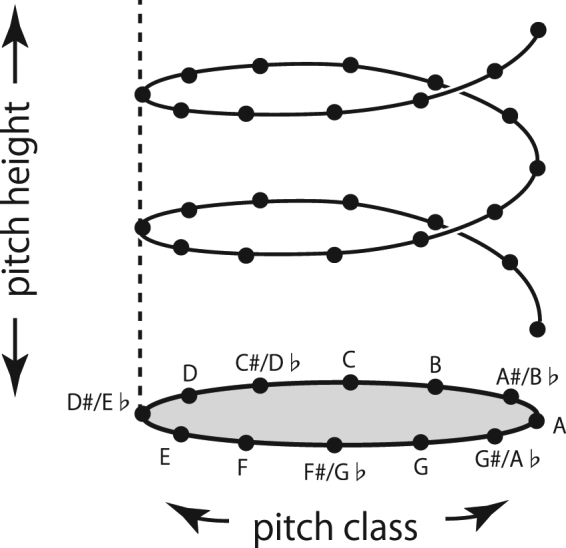
Helical model of musical pitch perception, in which pitch height and pitch class (or pitch chroma) represent the two psychological dimensions of pitch.

Musical pitch, or the perception of pitches in musical contexts, is not simply defined by tone frequency, because it is also constrained by the qualitative measure of sounds that reflect the concept of octave. The former aspect of musical pitch is referred to as *pitch height*, and the latter as *pitch class* or *pitch chroma*. Pitch height can be defined as a one-dimensional linear continuum from low frequency to high frequency and pitch chroma as a one-dimensional circle (C, C#, D, etc.). Therefore, musical pitch may be represented in a helical structure; this is illustrated in Fig. 1 ^1,2^. In sound-color synesthesia or anomalous color sensations produced by sounds, it is well known that high and low frequency sounds are associated with bright and dark colors, respectively, not only in people with synesthesia but also in those without it^3–7^. In contrast, how pitch class is linked to color in synesthetic sensations has not been explored adequately. Considering that musical pitch is composed of two components, pitch height and pitch class, we hypothesized that “pitch-color” synesthesia also has two components, namely, “pitch height-color” synesthesia and “pitch class-color” synesthesia. In this study, the first detailed experimental investigation of pitch class-color synesthesia was conducted.

In contrast to the rather universal correlation between pitch height and brightness, color sensations associated with a given pitch class appear to differ widely from individual to individual. This is illustrated by the well-known story of such disagreement between the two synesthetic composers, Nikolai Rimsky-Korsakov and Franz Liszt^8^. Thus, it is often believed that pitch class-color synesthesia is idiosyncratic, unlike pitch height-color synesthesia. However, this notion requires verification, considering the paucity of research conducted on this topic. Even in the case of grapheme-color synesthesia, where the correspondence between letters and colors is often considered idiosyncratic, a large cohort study suggested that some letters tend to be associated with certain specific colors, for example, “a” with red, “b” with blue, and “c” with yellow^9^. Accordingly, we systematically investigated whether there is any rule of how pitch classes are mapped to a three-dimensional color space, namely hue (H), saturation (S), and value/brightness (V), in fifteen pitch class-color synesthetic subjects.

## Materials and Methods

### Subjects

Fifteen subjects with pitch class-color synesthesia participated in the study (18–22 years old, two males; Table 1). All subjects reported that they had sensations of colors associated with pitch classes. The validity of their claim was confirmed by a consistency test, which is described below. Twelve subjects reported experiencing spontaneous color sensations when listening to music or musical sounds; that is, they had “colored hearing” in the general meaning of the term, whereas the other three did not experience such sensations.

**Table 1:** Subject profiles.

Case number	Age	Sex	Music training (ages)	AP score	Colored hearing	Answer to Question 1	Answer to Question 2
1	22	f	5–18	97	yes	syllable, pitch	painted colors on notes
2	22	f	2–22	100	yes	syllable, pitch	no idea
3	22	m	7–22	100	yes	pitch	no idea
4	21	f	5–21	86	yes	syllable	no idea
5	18	f	5–15	100	yes	syllable, pitch	no idea
6	21	f	6–20	100	yes	syllable	no idea
7	19	f	4–19	86	yes	syllable, pitch (inconsistent)	color stickers on keyboard
8	21	f	5–18	100	yes	syllable	color stickers on keyboard
9	20	f	4–20	100	yes	syllable	color stickers on keyboard
10	19	f	3–18	86	yes	pitch	no idea
11	19	f	11–19	100	yes	syllable	color stickers on keyboard
12	20	f	3–20	91	yes	syllable, pitch keyboard key	color stickers on keyboard
13	20	m	4–17	91	no	syllable	no idea
14	18	f	4–18	94	no	syllable	no idea
15	20	f	6–14	80	no	syllable	no idea

Question 1: What triggers your color sensation: pitch or syllable? Question 2: Do you have any idea how you acquired your synesthesia? Abbreviations: f, female; m, male.

An inclusion criterion for this study, other than having pitch class-color synesthesia, was that the subjects had to have moderate to high levels of absolute pitch (AP). Although we did encounter a number of pitch class-color synesthetes with no, or low levels of, AP, these subjects will be examined later in a separate study that will specifically address the effects of AP on pitch class-color synesthesia. The AP ability of subjects was evaluated by our AP test^10^, in which the subjects named the pitch class of 60 chromatic scale notes in piano timbre covering five octaves, presented randomly in sequence with a stimulus onset asynchrony of four seconds. The criterion was a score of 80% correct or higher (average ± s.d.: 94.1 ± 6.9%) for the white-key notes (*do*, *re*, *mi*, *fa*, *sol*, *la*, and *si*) that represented the seven base pitch classes with no accidentals (sharp or flat). AP is acquired through early music training^11,12^, and thus all participants in this study had received formal music training outside of standard school education; their average (± standard deviations, s.d.) number of years in training was 13.8 ± 3.2 years, and they had started at a mean age of 4.9 ± 2.1. In Table 1, the participants’ AP scores and their years of music training are presented.

All the subjects were undergraduate or graduate students of the University of Niigata, Japan, and were native Japanese speakers. The study was approved by the Internal Review Board of the University of Niigata and was carried out in accordance with the human research guidelines of the Internal Review Board of the University of Niigata. All subjects gave written informed consent before participating in the study.

### Color selection test

Subjects selected one color for each of the 12 pitch classes of the chromatic scale on typical color-selection software (FE-Color Palette, Fieldeast, Japan, http://www.fieldeast.com/soft/fecp.html), running on a computer that was connected to a cathode ray tube monitor (SONY G520). The pitch classes were specified verbally by the experimenter, who employed solfège syllables (*do*, *re*, *mi*, *fa*, *sol*, *la*, and *si*) and accidentals. In Japan where the work was conducted, pitch classes are commonly specified in this way rather than with letters (C, D, E, etc.) or other symbols/names. Not all synesthetic subjects had color sensations for all 12 pitch classes; in such case, they were not required to provide an answer.

To confirm consistency of the sensation, the test was administered on two different days separated by 196 ± 89 (mean ± s.d.) days. Furthermore, the test was administered twice on each day. The RGB (red, green, blue) values of the chosen colors were recorded, and the results were averaged across the repeated tests on each day, and, subsequently, across the two days. The order of the pitch classes was randomized for each test.

For numerical analyses of colors, normalized RGB values were converted to HSV values to represent them in hue (*H*), saturation (*S*), and value (*V*), by employing the following standard formulae:

1 $$H=(60\frac{(G-B)}{(MAX-MIN)}+360)\,\mathrm{mod}\,360\quad {\rm{if}}\,MAX=R,$$

2 $$H=60\frac{(B-R)}{MAX-MIN}+120\quad {\rm{if}}\,MAX=G,$$

3 $$H=60\frac{(R-G)}{MAX-MIN}+240\quad {\rm{if}}\,MAX=B,$$

4 $$H={\rm{undefined}}\quad {\rm{i}}{\rm{f}}\,MAX=MIN,$$

5 $$S=MAX-MIN,$$

6 $$V=MAX,$$

where *MAX* = max(*R*, *G*, *B*), and *MIN* = min(*R*, *G*, *B*). Finally, *H* (in degrees) was either converted to radians or to a normalized unit (NU, range of 0–1), whereas *S* and *V* were in NU.

### Phase unwrapping of hue

The values of *H*, calculated as shown above, posed a problem in numerical analyses of hue, because of its circularity. For example, a red hue, *H*_red_ = 0.01, and a violet hue, *H*_violet_ = 2π – 0.01, have quite different values of *H* despite their similar hues. Furthermore, their numerical mean is π (green) where it should be 0 (red). Therefore, the following adjustment was made to unwrap *H* in phase. First, the colors were averaged in the RGB space (either within or between subjects, depending on the case). Averaging in RGB preserved the colors as this operation was linear, unlike in the HSV space. Then, the averaged RGB values and the individual RGB values from which the average was derived were both converted to HSV to obtain averaged *H* (*H*_ave_) and individual *H* (*H*_ind_), respectively. Finally, each *H*_ind_ was unwrapped to be within the range of ± π from *H*_ave_, by utilizing the following conversion:

7 $${H}_{{\rm{ind}}({\rm{adjusted}})}={H}_{{\rm{ind}}}\quad {\rm{if}}\,{\rm{abs}}\,({H}_{{\rm{ind}}}\,\mbox{--}\,{H}_{{\rm{ave}}})\le {\rm{\pi }},$$

8 $${H}_{{\rm{ind}}({\rm{adjusted}})}={H}_{{\rm{ind}}}+2\pi \quad {\rm{if}}\,{\rm{abs}}\,({H}_{{\rm{ind}}}\,\mbox{--}\,{H}_{{\rm{ave}}}) > {\rm{\pi }}\,{\rm{and}}\,{H}_{{\rm{ave}}} > {\rm{\pi }},$$

9 $${H}_{{\rm{ind}}({\rm{adjusted}})}={H}_{{\rm{ind}}}-2{\rm{\pi }}\quad {\rm{if}}\,{\rm{abs}}\,({H}_{{\rm{ind}}}\,\mbox{--}\,{H}_{{\rm{ave}}}) > {\rm{\pi }}\,{\rm{and}}\,{H}_{{\rm{ave}}}\le {\rm{\pi }},$$

where abs indicates absolute value.

In the above example of *H*_red_ = 0.01 and *H*_violet_ = 2π − 0.01, the adjusted values of *H* would be 0.01 and −0.01, respectively, with their mean *H*_ave_ being 0.00. The range of adjusted *H* was −π to 3π. Unwrapped *H* values were used in all statistical analyses, unless otherwise noted.

### Behavior experiments

The majority of our subjects reported in interviews that, by introspection, their color sensations were linked to the names, that is, solfège syllables, of pitch classes rather than to their pitches (Table 1). A set of behavior experiments was conducted to test this notion. There were four tasks in which two types of stimuli, solfège syllable and pitch, were each presented with two different instructions, namely, to report the associated color or to report the solfège syllable (Table 2).

**Table 2:** Behavioral tasks.

		Response	
		Syllable (voice)	Color (voice)
Stimulus	Syllable (visual)	Syllable-to-syllable task	Syllable-to-color task
	Pitch (auditory)	Pitch-to-syllable task	Pitch-to-color task

The modality of stimulus/response is shown in parentheses.

These experiments were designed under the hypothesis that a two-step process underlies pitch class-color synesthesia in subjects with AP. Pitches were first converted to solfège syllables by using AP, which was an automatic process that occurred without conscious effort^10^. Subsequently, the solfège syllables were associated with colors by synesthesia. Two predictions followed this hypothesis. First, given a musical pitch stimulus, reaction times (RT) for reporting solfège syllables would be shorter than those for reporting colors. This was tested by comparing RTs in the pitch-to-syllable task and the pitch-to-color task. Second, RTs for reporting colors would be shorter when solfège syllables were presented than when pitches were presented. This was tested by comparing RTs between the syllable-to-color task and the pitch-to-color task.

The syllable-to-color task evaluated the accuracy and speed of pitch class-color association, without the possible confounding effects of pitch height. The seven pitch-class names of white-key notes (*do*, *re*, *mi*, *fa*, *sol*, *la*, and *si*) were randomly presented on a computer screen in the form of a solfège syllable (in Japanese characters), and subjects reported vocally the associated color as fast as possible. The syllables were printed in white letters against a black background. A response was considered correct when it matched the color that the subject himself/herself had selected in the color-selection test, earlier on the same day, and only RTs for correctly responded trials were analyzed.

In the syllable-to-syllable task, subjects simply read the solfège syllables aloud that were presented on the computer screen as described above. This task involved converting the visual representation of a syllable to its auditory representation before the response.

The pitch-to-color task evaluated the accuracy and speed of pitch class-color association, which is the defining feature of pitch class-color synesthesia. In the pitch-to-color task, a random sequence of white key notes covering two octaves (A3-G4) were presented in piano timbre tuned to A4 = 440 Hz. The subjects reported vocally the associated color they experienced (red, yellow, blue, etc. in Japanese) as fast as possible. A response was considered correct when it matched the color that the subject himself/herself had selected in the color-selection test, earlier on the same day, and only RTs for correctly responded trials were analyzed.

In the pitch-to-syllable task, subjects reported the pitch class of piano tones that were presented as described above. This condition evaluated the accuracy and speed of pitch-to-syllable association, or pitch class identification; this was the defining feature of AP.

Assuming that the syllable-to-color task involved a two-step process in which the visual representation of the syllable was first converted to its auditory representation and then to a color, the difference in RTs between the syllable-to-color task and the syllable-to-syllable task, ΔRT_syllable_ = RT_syllable-to-color_ − RT_syllable-to-syllable_, would provide an assessment of the time required for the second step: syllable (auditory)-to-color mapping. On the other hand, under the hypothesis that pitches were first converted to syllables before they evoked color sensations, the difference in RTs between the pitch-to-color task and the pitch-to-syllable task, ΔRT_pitch_ = RT_pitch-to-color_ − RT_pitch-to-syllable_, provided another estimate of the time required for syllable (auditory)-to-color conversion.

In all tests, subjects were asked to respond vocally as fast as possible. RTs were measured by using voice onsets. The order of the tasks was randomized across subjects. In each task, each pitch class was presented 10 times in a randomized order and results were averaged across all trials and pitch classes. The experiments were conducted using Presentation computer software (Neurobehavioral Systems, Berkeley, CA, USA) and Sound Blaster audio hardware (Creative Technology, Jurong East, Singapore).

**Figure 2:**
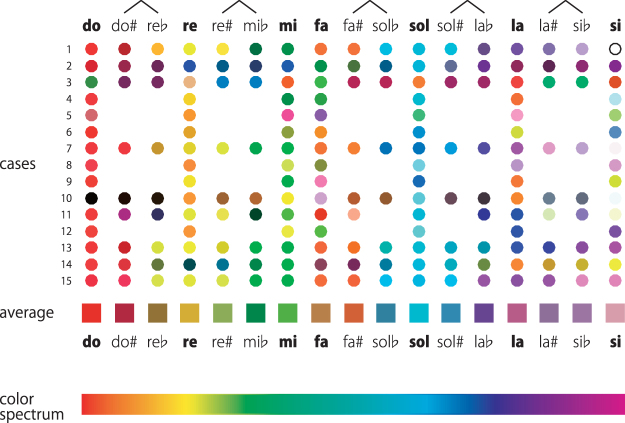
Results of the color-selection test. Colors selected by individual subjects are shown as circles arranged in rows and the averaged colors are shown with squares. An open circle denotes the color white, whereas a missing circle indicates that the subject had no colors associated with that pitch class. Linked pitch classes represent enharmonic equivalents. The case numbers correspond to those in Table 1.

It was possible that the visual presentation of syllables interfered with the task by facilitating answering “white,” for example. This was a limitation of the study. However, only a limited number of subjects had a white color sensation associated with pitch classes (Fig. 2), and such effects were assumed to be negligible.

### Statistical analyses

IBM SPSS Statistics version 22 (IBM, Armonk, NY, USA) was used to perform the statistical calculations. Models of statistical analyses are described in the section on results when relevant.

### Data availability

The datasets generated during and/or analyzed during the current study are available from the corresponding author on reasonable request.

## Results

### Pitch class-color mapping

Figure 2 depicts the colors selected by all individual subjects, as well as their averaged colors. Despite the presence of individual differences, clear tendencies for some pitch classes to be associated with certain specific colors existed, for example *do* with red, *re* with yellow, and *sol* with cyan. Further examinations revealed two main observations.

First, the colors of enharmonic pitch classes were affected by how they were named. For example, although both referred to the same note, for many subjects, *do-sharp* had a reddish color whereas *re-flat* was yellowish. Similarly, the averaged color for *sol-sharp* was more bluish than *la-flat*, which was purplish. Furthermore, such differentiation of hue was evident in other enharmonic notes. In other words, the colors were linked to the verbal labels of pitch classes rather than the actual auditory pitches. These observations were further supported by behavioral data, which are presented below.

**Figure 3:**
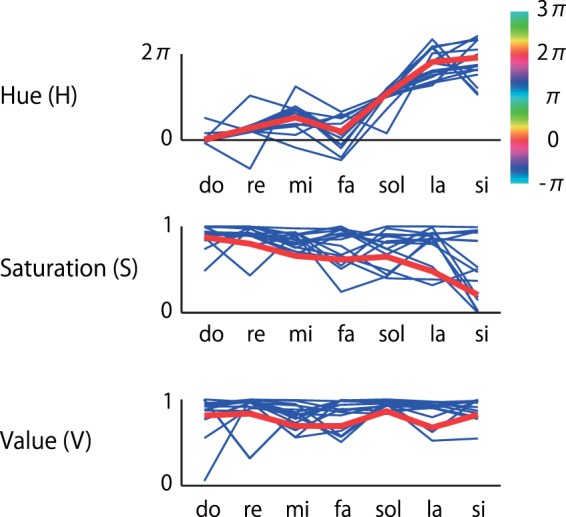
Numerical representations of the colors in Fig. 2, plotted separately for hue, saturation, and value dimensions of color in the HSV space. Each blue line represents a single case, and the red line represents the average color. The unit of hue is in radians (after phase unwrapping; see methods). Saturation and value are in a normalized unit.

Second, the overall pattern of color changes across the pitch classes resembled the color spectrum of a rainbow (Fig. 2). The colors were converted to the HSV space to analyze the relationship between pitch class and the three dimensions of color perception, namely, hue (*H*), saturation (*S*), and value (*V*), numerically (Fig. 3). Only the white-key notes were included in the following analyses because not all subjects had color sensations associated with black-key notes. As a result, *H* increased in value from approximately 0 (in radian, representing red) to nearly 2π (violet) in an approximate linear manner, as the pitch class rotated in phase from *do* to *si*. In other words, there appeared to be a simple approximate relationship, *hue* = *pitch class*, when the variables *hue* and *pitch class* were both expressed in radians, with 0 radian corresponding to red (in hue) or *do* (in pitch class). This hypothesis was tested by a regression analysis with a random intercept and slope for each subject; the dependent variable was hue and the repeated-measures random-effect variable was pitch class (0.00, 0.90, 1.80, 2.70, 3.59, 4.49, and 5.39 radians corresponding to *do* through *si*). As a result, the estimated slope of regression was 1.09 (95% confidence interval [CI] 0.94–1.25), *t*(130.8) = 14.1, *p* < 0.001), which indicated that the relationship *hue* = *pitch class* represented a good first approximation. There was, however, an apparent deviation from this simple relationship at *fa*, the significance of which requires future examination (Figs 2 and 3).

Regarding the other dimensions of color, saturation (S) gradually decreased as the pitch class rotated in phase from *do* to *si*, such that the colors were less colorful for *la* and *si* in comparison with to *do*, *re*, and *mi* (Figs 2 and 3), although this effect was not as evident as the above relationship between pitch class and hue. Statistically, the regression analysis revealed that the correlation was significant with an estimated slope of −0.36 (95% CI −0.50– −0.22), *t*(101.5) = 5.2, *p* < 0.001.

### Consistency within subjects

**Figure 4:**
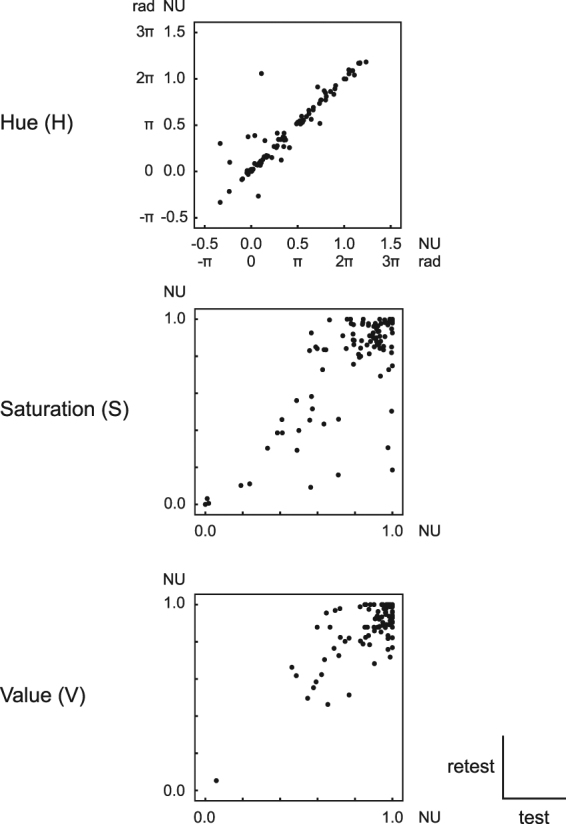
Test–retest consistency of color reports. The reported colors were consistent between the test and retest, particularly regarding hue. NU: normalized unit.

Within-subject consistency is an important objective criterion for proving synesthetic sensations. In our study, subjects selected colors for pitch classes on two occasions separated on average by as long as three months, and the test–retest scatter plot of HSV values indicated a high reliability of their color sensations, particularly concerning hue (Fig. 4).

A linear regression analysis with random intercept and slope for each subject, using a compound symmetry covariance matrix, was performed to evaluate how well the HSV values in the first test predicted the HSV values in the retest. The dependent variable was the HSV values for the seven pitch classes, which was obtained in the second test, and the HSV values in the first test served as the independent random variable. The slope of regression would be one if the results of two tests were identical and zero if random. The mean slope of regression for hue (H) was found to be 0.92 (95% CI 0.84–0.99), *t*(101.5) = 24.9, *p* < 0.001. For saturation (S), the slope was 0.89 (95% CI 0.75–1.03), *t*(12.8) = 21.5, *p* < 0.001, and the mean slope of regression for the value (V) dimension was 0.67 (95% CI 0.46–0.89), *t*(11.9) = 6.8, *p* < 0.001.

### Behavior experiments

**Figure 5:**
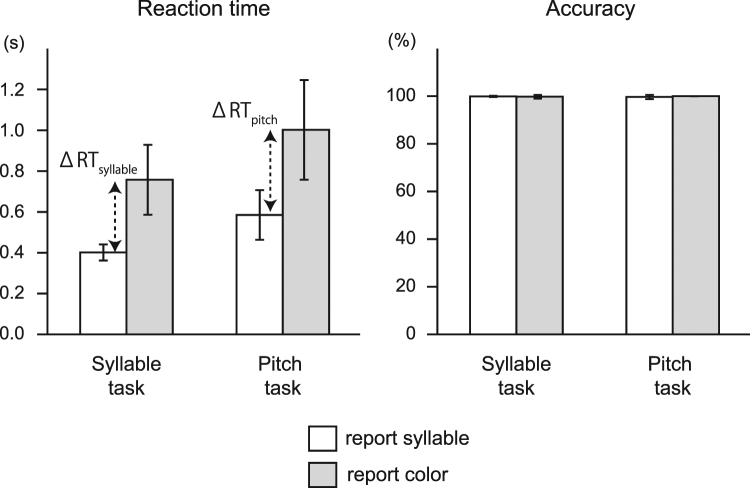
Behavioral results. Subjects performed nearly perfectly in terms of accuracy, confirming their AP (pitch-to-syllable task) and synesthesia (pitch-to-color task and syllable-to-color task). RTs were shorter in the syllable task than in the pitch task and for reporting syllables than for reporting colors. The differences in RTs between reporting a color and reporting a syllable (ΔRT_syllable_ in the syllable task and ΔRT_pitch_ in the pitch task) provided estimates of the time required for syllable-to-color association. Error bars indicate standard deviations.

The results of the behavior experiments are shown in Fig. 5. The subjects exhibited perfect or near-perfect scores in all tasks in terms of accuracy, thus confirming their possession of AP (pitch-to-syllable task) and synesthesia (pitch-to-color task and syllable-to-color task). Analyses of RTs disclosed two observations that supported the hypotheses that colors were more closely associated with pitch-class names than with pitches, and that the pitch-to-color association in our subjects involved a two-step process in which pitches were first converted to pitch-class names, and then the pitch-class names were associated with colors. In other words, the speed for reporting colors was faster in the syllable-to-color task than in the pitch-to-color task, and RTs were shorter in the pitch-to-syllable task than in the pitch-to-color task. These results were supported statistically, as detailed below.

The RT data were analyzed by a repeated-measures analysis of variance (ANOVA) with factors, task (pitch task/syllable task), and response (report pitch/syllable). The main effect of task and the main effect of response were found to be significant, *F*(1, 14) = 53.9, *p* < 0.001 and *F*(1, 14) = 79.9, *p* < 0.001, respectively, whereas the interaction was not significant, *F*(1, 14) = 3.3, *p* = 0.090. In other words, the responses were generally faster in the syllable task than in the pitch task, irrespective of the type of response, and the responses were generally faster for reporting syllables than for reporting colors, irrespective of the type of stimulus.

The difference in RTs between the syllable-to-color task and the syllable-to-syllable task, or ΔRT_syllable_, provided an estimate of the time required for syllable-to-color conversion (Fig. 5). The average of ΔRT_syllable_ was 356 ± 159 ms. Similarly, the difference in RTs between the pitch-to-color task and the pitch-to-syllable task, or ΔRT_pitch_, of 416 ± 198 ms, provided another measure of the time required for syllable-to-color conversion. These two estimates were not affected by the type of stimulus. Statistically, the one-way repeated-measures ANOVA with task (syllable task/pitch task) as a factor revealed that ΔRT_syllable_ and ΔRT_pitch_ were comparable, *F*(1, 14) = 3.3, *p* = 0.090.

## Discussion

The study provided the first detailed experimental investigation into “pitch class-color synesthesia,” which has been clearly distinguished in definition and phenomenon from the well-described crossmodal association between pitch height and brightness/lightness. Two main findings advanced the current understanding on how musical pitches are represented across sensory modalities in the human brain.

First, pitch classes had rainbow hues in pitch class-color synesthesia (Figs 2 and 3). Despite the presence of substantial individual differences, there nevertheless was a systematic, but probably latent, crossmodal correspondence between pitch class and the hue dimension of color. Pitch class was also negatively correlated with saturation, such that *la* and *si* were less saturated (i.e., less colorful and more muted) than *do*, *re*, and *mi*. However, this effect was not as evident as the relationship with hue. The cause of the rainbow color mapping remains unclear. Interviews with our subjects revealed that the majority of them (9/15) had no idea how they acquired their color sensations; this is typical of synesthesia (Table 1). The others recalled that when they started to learn the piano, typically at preschool ages, their music teachers used colored stickers to label keys on the keyboard (five subjects), or they were made to paint notes with colors on printed music (one subject). None of them, however, reported that the colors designated by the teacher had rainbow hues. The effects of musical experiences on pitch class-color synesthesia remain inconclusive and require further investigation.

Of great importance, the synesthetic colors associated with pitch classes did not vary in their value/brightness. This is consistent with our premise that given the two-component helical model of musical pitch perception, pitch-color synesthesia also comprises two components, namely pitch height-color synesthesia and pitch class-color synesthesia. Combined with the previously reported crossmodal correlation between pitch height and brightness/lightness^3,4,6,7^, the findings suggest a new model of pitch-color synesthesia, in which the pitch-height dimension of pitch correlates with value/brightness, and the pitch-class dimension maps to the hue and saturation dimensions of color in the HSV space.

Second, synesthetic color sensations in pitch class-color synesthesia were associated more directly with pitch-class names than with the sounds of the notes. Pitch class is a higher-order attribute of pitch that is obtained by discretizing the pitch continuum at semitone steps, accompanied by octave generalization that defines the helical structure of pitch perception (Fig. 1). As listeners learn to recognize pitch classes through music training, such categorization of pitch is often, if not always, accompanied by verbal labeling of pitch classes. It was probably at this level of verbal representation that pitch classes were linked to colors. In fact, the majority of our synesthetic subjects (13/15) reported explicitly that their colors were linked to solfège syllables, although five of them claimed that their color sensations could also be evoked by pitches or keys on a keyboard (Table 1). However, two subjects claimed that pitches directly evoked colors. It is possible that the processes underlying pitch class-color synesthesia are not identical among different synesthetes.

In contrast to the traditional view of synesthesia that one sensation is associated with another sensation at a low, pre-attentive level of sensory processing^13^, it has become evident that higher-level concepts or meaning can also induce synesthetic experiences, which are referred to as ideasthesia^14^. In ideasthesia, it is the extracted meaning of a stimulus rather than the physical properties of the sensory input that triggers synesthesia. For example, a shape stimulus can evoke different color sensations depending on whether it is interpreted as either the letter Z or the number 2^15^. Pitch class-color synesthesia is an example of ideasthesia in that colors are associated with the concept or the verbal label of pitch class. Pitch class, in contrast to pitch height, is a higher-level attribute of sound that is learned and elaborated through music education^16,17^. Therefore, acquisition of pitch class-color synesthesia is expected to require a certain amount of music training, at least to a level that the concept of pitch classes is learned. All of our synesthetic subjects had in fact received at least eight years, typically over 10 years, of formal music training outside standard school education; they often started their music training at preschool ages (Table 1). This is in sharp contrast to the crossmodal association between pitch height and brightness/lightness, which requires no specific music training and is even seen in non-human primates^18^. This is another rationale for distinguishing pitch class-color synesthesia and pitch height-color synesthesia.

Various attributes of sound such as pitch height, timbre, tonality, and loudness can evoke synesthetic color sensations^19^. It is almost certain that these various types of synesthesia have differentiated underlying mechanisms that reflect how these sound attributes are represented in the human auditory system. The present work defined and provided the first detailed descriptions of pitch class-color synesthesia. A remarkable feature of pitch class-color synesthesia was that the colors were associated with the verbal labels of pitch classes in a systematic manner such that the solfège syllables had rainbow hues. The conversion from pitch-class name to color was a fast process that took less than 500 ms. Because of the significant role of verbal labels on pitch class-color association, it is possible that differences in language or a pitch-naming system lead to different color mapping schemes. In addition, environmental factors such as music education may also affect the condition. Multicultural studies are needed to test the universality of our findings.
